# Associations between nutrients in one-carbon metabolism and fetal DNA methylation in pregnancies with or without gestational diabetes mellitus

**DOI:** 10.1186/s13148-023-01554-1

**Published:** 2023-08-26

**Authors:** Isma’il Kadam, Mudar Dalloul, Jeanette Hausser, Monique Huntley, Lori Hoepner, Lawrence Fordjour, Joan Hittelman, Anjana Saxena, Jia Liu, Itamar D. Futterman, Howard Minkoff, Xinyin Jiang

**Affiliations:** 1 Departments of Health and Nutrition Sciences, Brooklyn College of City University of New York, 2900 Bedford Ave, Brooklyn, NY 11210 USA; 2 Department of Obstetrics and Gynecology, State University of New York Downstate Health Sciences University, Brooklyn, NY 11203 USA; 3 Department of Environmental and Occupational Health Sciences, State University of New York Downstate Health Sciences University, Brooklyn, NY 11203 USA; 4 Department of Pediatrics, State University of New York Downstate Health Sciences University, Brooklyn, NY 11203 USA; 5 Department of Psychology, State University of New York Downstate Health Sciences University, Brooklyn, NY 11203 USA; 6 Departments of Biology, Brooklyn College of City University of New York, Brooklyn, USA; 7 Neuroscience Initiative, Advanced Science Research Center at the Graduate Center of the CUNY, New York, NY 10031 USA; 8 Division of Maternal Fetal Medicine, Departments of Obstetrics and Gynecology, Maimonides Medical Center, Brooklyn, USA

## Abstract

**Background:**

Gestational diabetes mellitus (GDM), characterized by hyperglycemia that develops during pregnancy, increases the risk of fetal macrosomia, childhood obesity and cardiometabolic disorders later in life. This process has been attributed partly to DNA methylation modifications in growth and stress-related pathways. Nutrients involved with one-carbon metabolism (OCM), such as folate, choline, betaine, and vitamin B_12_, provide methyl groups for DNA methylation of these pathways. Therefore, this study aimed to determine whether maternal OCM nutrient intakes and levels modified fetal DNA methylation and in turn altered fetal growth patterns in pregnancies with and without GDM.

**Results:**

In this prospective study at a single academic institution from September 2016 to June 2019, we recruited 76 pregnant women with and without GDM at 25–33 weeks gestational age and assessed their OCM nutrient intake by diet recalls and measured maternal blood OCM nutrient levels. We also collected placenta and cord blood samples at delivery to examine fetal tissue DNA methylation of the genes that modify fetal growth and stress response such as insulin-like growth factor 2 (*IGF2*) and corticotropin-releasing hormone (*CRH*). We analyzed the association between maternal OCM nutrients and fetal DNA methylation using a generalized linear mixed model. Our results demonstrated that maternal choline intake was positively correlated with cord blood *CRH* methylation levels in both GDM and non-GDM pregnancies (*r* = 0.13, *p* = 0.007). Further, the downstream stress hormone cortisol regulated by CRH was inversely associated with maternal choline intake (*r* = − 0.36, *p* = 0.021). Higher maternal betaine intake and serum folate levels were associated with lower cord blood and placental *IGF2* DNA methylation (*r* = − 0.13, *p* = 0.049 and *r* = − 0.065, *p* = 0.034, respectively) in both GDM and non-GDM pregnancies. Further, there was an inverse association between maternal betaine intake and birthweight of infants (*r* = − 0.28, *p* = 0.015).

**Conclusions:**

In conclusion, we observed a complex interrelationship between maternal OCM nutrients and fetal DNA methylation levels regardless of GDM status, which may, epigenetically, program molecular pathways related to fetal growth and stress response.

**Supplementary Information:**

The online version contains supplementary material available at 10.1186/s13148-023-01554-1.

## Background

The epigenome, such as methylation of DNA, controls gene expression without altering the DNA sequence [1]. The epigenome of the fetus is susceptible to modifications by intrauterine exposures such as nutrition, toxins or even seasonality [2]. These early-life alterations in the epigenome may have long-lasting effects on gene expression and functioning of various molecular pathways in later life [2, 3]. Gestational diabetes mellitus (GDM), hyperglycemia that arises during pregnancy in previously euglycemic individuals [4], has been demonstrated to alter the placental and fetal epigenome, serving as a mechanism to result in fetal overgrowth or macrosomia that persists to obesity in childhood and cardiometabolic dysfunction later in life [5–11]. The insulin-like growth factor (IGF) pathway is a major fetal and placental growth-promoting pathway modified by DNA methylation of its components such as IGF2 and IGF binding proteins and is associated with fetal overgrowth in GDM [12, 13]. In addition, the hypothalamic–pituitary–adrenal (HPA) axis mediates response to intrauterine stress, including hyperglycemic stress, and has demonstrated either a positive or a negative association with birth weight in different studies [14, 15]. Chronic elevation of the effector hormone in this axis, cortisol, is associated with hypertension, type 2 diabetes mellitus, and mental health issues [16]. Promoter DNA methylation of the upstream hormone in this axis, corticotropin-releasing hormone (CRH), is susceptible to intrauterine environment alteration, which may then affect HPA axis activity and eventually fetal growth and cardiometabolic disease risk in the long term [16–18].

Dietary interventions among pregnant women with GDM improve maternal weight management and blood glucose control, yet cannot completely prevent fetal macrosomia [19–23]. It is also largely unknown how to restore the fetal epigenome that is modified by the hyperglycemic intrauterine environment, thereby reducing the risk of obesity and cardiometabolic disorders later in life. Nutrients involved with one-carbon metabolism (OCM) in the methionine cycle supplies the methyl groups necessary for DNA methylation [24], which may interact with maternal GDM status in determining the fetal epigenome.

In the methionine cycle, both 5-methyl tetrahydrofolate (THF) and choline (after oxidation to betaine) donate their methyl groups to homocysteine and remethylate it to methionine. The reaction of 5-methyl THF requires vitamin B_12_ as a coenzyme for methionine synthase. Methionine is converted to* S*-adenosylmethionine (SAM), the universal methyl donor which provides its methyl group to DNA, protein, and lipid during methylation reactions. SAM converts to *S*-adenosylhomocysteine (SAH) after losing the methyl group and SAH is reversibly converted to homocysteine which completes the cycle [24]. Maternal intake of a methyl donor-enriched diet led to higher methylation and suppression of the *agouti* gene in the viable yellow agouti (A^vy^) mice, thereby eliminating the effect of this gene on offspring fur coat color, food intake, and obesity [25, 26]. In humans, however, the influence of methyl nutrients on the fetal epigenome seems to be complex and time-specific. For example, methyl nutrient intake or blood level has been associated with varied outcomes as there are reports suggesting increases, decreases or no changes in global methylation and *IGF2* differentially methylated region (DMR) methylation in cord blood at birth and saliva samples in early childhood [27–29]. In a randomized controlled trial (RCT) of choline supplementation during the 3rd trimester of pregnancy, a higher maternal choline intake led to higher *CRH* promoter methylation in the placenta, but reduced methylation of *CRH* in the cord blood [30]. It is largely unknown whether increasing maternal methyl nutrient intake and blood levels can rescue GDM-mediated alterations in fetal DNA methylation.

In this prospective study, we examined how maternal methyl nutrient intake and serum levels in pregnancies with and without GDM interact with global and site-specific DNA methylation of *IGF2* and *CRH* in the placenta and cord blood.

## Results

### Maternal choline intake and plasma levels differed between GDM and non-GDM pregnancies

We have previously reported the demographic information of this prospective observational cohort that enrolled pregnant women with and without GDM at 25–33 weeks gestational age during active rolling enrollment [31]. Since the last report [31], we recruited additional participants making a total 40 participants with GDM and 36 participants without GDM in the current study. Placenta and cord blood samples were collected at delivery and available in 21 GDM and 26 non-GDM participants. Updated demographic information is presented in Additional File 1. Table S1.

In an unadjusted analysis of covariance (ANCOVA) model (Model 1) and a model adjusted for demographic differences (Model 2), total folate intake was lower in GDM versus non-GDM participants (*p* = 0.04) (Table 1). However, after further adjusting for total energy intake and prepregnancy BMI as a proxy of energy requirement in Model 3, the intake comparison between groups was no longer significant (*p* = 0.9). In addition, only in Model 3 choline intake was significantly higher (*p* = 0.001) in GDM versus non-GDM (Table 1). Betaine, natural food folate, and vitamin B_12_ intakes were similar between the two groups. Maternal plasma free choline levels were higher in GDM versus non-GDM in all models (*p* values = 0.01–0.02) (Table 1). Other metabolites including plasma betaine, homocysteine, as well as serum holotranscobalamin (holoTC, a marker of vitamin B_12_ status) and folate did not differ by GDM status.

**Table 1:** Maternal methyl nutrient intake and blood level in GDM and non-GDM participants

	GDM	non-GDM	Model 1 *p*	Model 2 *p*	Model 3 *p*
Choline intake (mg/d)	433 ± 30	357 ± 28	0.94	0.88	0.001
Betaine intake (mg/d)	131 ± 16	113 ± 14	0.28	0.31	0.12
Folate intake (µg/d)	435 ± 45	431 ± 41	0.01	0.04	0.90
Vitamin B_12_ intake (µg/d)	5.6 ± 1.5	4.9 ± 1.4	0.98	0.95	0.57
Dietary methylation score	10.7 ± 0.8	9.7 ± 0.7	0.28	0.38	0.12
Plasma free choline (μmol/L)	10.4 ± 1.0	8.6 ± 1.0	0.02	0.01	0.01
Plasma betaine (μmol/L)	12.8 ± 1.0	12.8 ± 1.1	0.94	0.49	0.89
Serum folate (μg/L)	19.3 ± 2.8	15.6 ± 2.7	0.95	0.24	0.10
Serum holotranscobalamin (μg/L)	16.1 ± 1.2	16.5 ± 1.2	0.62	0.63	0.70
Plasma homocysteine (mmol/L)	10.7 ± 3.0	9.2 ± 3.0	0.72	0.34	0.48
Cord DNA methylation (Fold difference)	0.8 ± 0.1	1.0 ± 0.1	0.06	0.24	0.16
Placental DNA methylation (Fold difference)	0.8 ± 0.1	1.0 ± 0.1	0.11	0.46	0.54
Cord plasma SAM/SAH	0.07 ± 0.05	0.08 ± 0.04	0.93	0.61	0.81
Cord *CRH* methylation (%)	60 ± 1	52 ± 2	0.43	0.34	0.001
Placenta *CRH* methylation (%)	27 ± 3	26 ± 2	0.54	0.53	0.75
Cord *IGF2* methylation (%)	27 ± 4	27 ± 5	0.57	0.68	0.97
Placenta *IGF2* methylation (%)	33 ± 3	33 ± 5	0.78	0.54	0.79

*n* = 40 for the GDM group and *n* = 36 for non-GDM group for maternal biomarkers; *n* = 21 for the GDM group and *n* = 26 for the non-GDM group for placental and cord blood markers. Analyzed with ANCOVA except for average *IGF2* and *CRH* methylation which were analyzed with the generalized linear mixed model; for maternal measurements, Model 1 did not adjust for covariates; Model 2 adjusted for maternal age, parity, race/ethnicity, and maternal education levels; Model 3 adjusted for pre-pregnancy BMI and total energy intake in addition to covariates in Model 2. For cord blood and placental measurements, neonate sex, gestational age at birth, and mode of delivery were also included as covariates in model 2 and 3. Values are mean ± standard deviation (SD). *CRH* corticotropin-releasing hormone, *IGF2* insulin-like growth factor 2, *SAM* *S*-adenosylmethionine, *SAH* *S*-adenosylhomocysteine

The cord blood SAM/SAH ratio, a potential indicator of methylation potential, did not differ by GDM status. We also explored the global DNA methylation and site-specific methylation of *IGF2* (mediating fetal growth) DMR (a region homologous to the mouse DMR0 that locates upstream of promoter 3) and *CRH* (a key component in the HPA axis of stress response) promoter in placental and cord blood samples. In the full model (Model 3), average *CRH* promoter methylation (including CpG1, 2, 8, 9) levels in the cord blood were higher (*p* = 0.01) in the GDM versus non-GDM pregnancies (Table 1). There were no other differences in placental or cord blood global or site-specific methylation in GDM versus non-GDM pregnancies.

### Maternal transcobalamin and homocysteine levels were inversely associated with placental global DNA methylation

There were significant interactions between GDM status and maternal intake of betaine, vitamin B_12_, as well as the composite dietary methylation score (DMS) which assessed the overall intake of methyl nutrients in their associations with placental global DNA methylation (*p* = 0.003–0.01). Therefore, we stratified the data by GDM status, but did not find any significant association between these methyl nutrients and global placental DNA methylation in either GDM or non-GDM pregnancies (Table 2). Choline and folate intakes were also not related to placental DNA methylation. Both maternal holoTC (*r* = − 0.25, *p* < 0.01) and homocysteine (*r* = − 0.22, *p* < 0.01) levels were negatively associated with placental DNA methylation. Folate and choline metabolite levels in maternal blood were not associated with placental DNA methylation.

**Table 2:** Associations between maternal methyl nutrient intake or blood levels and global DNA methylation in the placenta and cord blood

	Placenta		Cord blood	
	*r*	*p*	*r*	*p*
Intake				
Choline	− 0.04	0.8	− 0.06	0.58
Betaine				
GDM	0.17	0.66	− 0.07	0.52
Non-GDM	− 0.31	0.16		
Vitamin B_12_				
GDM	− 0.006	0.87	0.17	0.29
Non-GDM	0.31	0.06		
Folate	− 0.22	0.1	0.01	0.96
Dietary methylation score				
GDM	− 0.11	0.41	0.04	0.85
Non-GDM	0.24	0.18		
*Blood metabolite*				
Choline	− 0.42	0.47	0.16	0.93
Betaine	− 0.47	0.55	0.01	0.96
Holotranscobalamin	− 0.25	0.007	0.05	0.99
Folate	0.09	0.47	0.07	0.73
Homocysteine				
GDM	− 0.22	< 0.01	0.24	0.62
non-GDM			0.49	0.01

*n* = 21 for the GDM group and *n* = 26 for the non-GDM group. Analyzed with generalized linear model adjusted for GDM status, GDM × nutrient intake/status interaction, total energy intake, and fetal covariates including sex of neonate, gestational age at birth, and mode of delivery. The correlation coefficient (*r*) was calculated using partial correlation controlling for the variables mentioned above

There was an interaction between maternal plasma homocysteine levels and GDM status in correlation with cord blood global DNA methylation (*p* = 0.03). After stratification by GDM status, a positive relationship (r = 0.49, *p* < 0.01) was found in non-GDM but not GDM pregnancies. Cord blood global DNA methylation was not associated with any other maternal nutrient intake or status markers.

### A higher maternal betaine intake was associated with lower cord blood IGF2 DMR methylation, placental IGF2 expression, and birth weight

We also examined the association of maternal methyl nutrients with placental and cord blood site-specific DNA methylation of loci identified as susceptible to modification by maternal methyl nutrients. The *IGF2* gene is an imprinted gene encoding the IGF2 protein that plays a critical role in placental and fetal growth during gestation [32]. We examined its methylation levels at the DMR0 upstream of the promoter in the placenta (including CpG1, 2, 3, 4, 5, 6–7, 8) since methylation of this region partly determines *IGF2* expression [33]. Maternal serum folate levels were inversely associated with the average methylation level of *IGF2* DMR0 (*r* = − 0.065, *p* = 0.034) while other methyl nutrient intake/status and the composite DMS did not have such an association (Table 3). When examining individual CpG sites, maternal vitamin B_12_ intake was positively associated with CpG1, 3, and 6–7 methylation (*r* = 0.22–0.44, *p* < 0.05) of this region (Additional File 2. Table S2). Additionally, maternal plasma betaine levels were positively associated with CpG6-7 methylation (*r* = 0.32, *p* = 0.028), while maternal serum folate levels were inversely associated with CpG5 (*r* = − 0.37, *p* = 0.01) and CpG8 (*r* = − 0.21, *p* = 0.014) methylation.

**Table 3:** Associations between maternal methyl nutrient intake or blood levels and *IGF2* DMR0 DNA methylation in the placenta and cord blood

	Placenta *IGF2* DMR0		Cord *IGF2* DMR0	
	*r*	*p*	*r*	*p*
Intake				
Choline	0.004	0.93	0.11	0.53
Betaine	− 0.11	0.89	− 0.13	0.049
Vitamin B_12_				
GDM	0.1	0.17	0.001	0.43
Non-GDM			0.21	0.12
Folate				
GDM	− 0.011	0.39	− 0.22	0.18
Non-GDM			0.077	0.9
Dietary methylation score	0.031	0.71	0.002	0.95
Blood metabolite				
Choline	0.017	0.66	0.06	0.45
Betaine	0.063	0.19	− 0.038	0.33
Holotranscobalamin	0.012	0.59	0.037	0.63
Folate	− 0.065	0.034	0.009	0.59
Homocysteine	0.051	0.41	0.075	0.73

*n* = 21 for the GDM group and *n* = 26 for the non-GDM group. Analyzed with generalized linear mixed model adjusted for GDM status, GDM × nutrient intake/blood status interaction, total energy intake, and fetal covariates including sex of neonate, gestational age at birth, and mode of delivery. The correlation coefficient (*r*) was calculated using partial correlation controlling for the variables mentioned above. *DMR0* differentially methylated region 0, *IGF2* insulin-like growth factor 2

As for cord *IGF2* DMR0 CpG methylation (including CpG1, 2, 3, 4, 6–7, and 8), maternal betaine intake was inversely associated with average DNA methylation of the region (*r* = − 0.13, *p* = 0.049) (Table 3). When examining methylation of individual CpG sites, maternal betaine intake was inversely associated with methylation of CpG4 (*r* = − 0.30, *p* = 0.013) and CpG8 (*r* = − 0.26, *p* = 0.038), whereas maternal choline and B_12_ intakes were positively associated with CpG2 (*r* = 0.38, *p* = 0.007) and CpG1 (*r* = 0.32, *p* = 0.038) methylation, respectively (Additional file 2: Table S2). Maternal plasma choline levels were positively associated with CpG6-7 (*r* = 0.31, *p* = 0.001) methylation levels.

Since IGF2 plays an important role in regulating placental and fetal weight, we assessed the association between these weights and maternal methyl nutrient intake and status. We found that maternal betaine intake was negatively associated with birthweight of infants (*r* = − 0.28, *p* = 0.015). We also examined the expression of *IGF2* mRNA in the placenta and found it to be negatively associated with maternal betaine intake levels as well (*r* = − 0.31, *p* = 0.038). However, there was no association between maternal betaine intake and placental weight (average 530 ± 140 g, *r* = − 0.069, *p* = 0.66) or cord blood IGF2 protein levels (average 6.7 ± 27.5 ng/mL, *r* = − 0.12, *p* = 0.56). GDM status did not modify the above associations.

### A higher maternal choline intake was associated with higher CRH promoter methylation and lower cortisol levels in the cord blood

We also examined placenta and cord DNA methylation of *CRH* promoter. None of the methyl nutrient intake/blood level was associated with average methylation of this region in the placenta (including CpG1, 2, 4, 8, 9, and 10) (Table 4). As for individual CpG sites, maternal betaine and B_12_ intakes, the composite DMS, and plasma betaine levels were all positively associated with DNA methylation at CpG8 (*r* = 0.34–0.36, *p* = 0.009–0.034) (Additional file 3: Table S3). However, serum folate and holoTC levels were negatively associated with CpG2 methylation (*r* = − 0.38 and − 0.33, *p* = 0.011 and 0.033).

**Table 4:** Associations between maternal methyl nutrient intake or blood levels and *CRH* promoter DNA methylation in the placenta and cord blood

	Placenta *CRH* methylation		Cord *CRH* methylation	
	*r*	*p*	*r*	*p*
Intake				
Choline	0.011	0.4	0.13	0.007
Betaine	0.067	0.79	− 0.003	0.74
Vitamin B_12_	0.082	0.32	− 0.004	0.39
Folate	0.094	0.21	0.063	0.55
Dietary methylation score	0.092	0.61	0.047	0.22
Blood metabolite				
Choline	0.001	0.91	− 0.018	0.44
Betaine	0.15	0.067	0.045	0.3
Holotranscobalamin	− 0.061	0.29	0.074	0.04
Folate	− 0.047	0.67	0.021	0.6
Homocysteine	− 0.09	0.62	0.08	0.39

*n* = 21 for the GDM group and *n* = 26 for the non-GDM group. Analyzed with generalized mixed model adjusted for GDM status, GDM × nutrient intake/blood status interaction, total energy intake, and fetal covariates including sex of neonate, gestational age at birth, and mode of delivery. The correlation coefficient (*r*) was calculated using partial correlation controlling for the variables mentioned above. *CRH* corticotropin-releasing hormone

Both maternal choline intake (*r* = 0.13, *p* = 0.007) and serum holoTC (*r* = 0.074, *p* = 0.04) were positively associated with average *CRH* DNA methylation in the cord blood (including CpG1, 2, 8, and 9). As for individual CpG sites, maternal choline intake was positively associated with methylation of CpG1(*r* = 0.55, *p* = 0.008) and CpG2 (*r* = 0.46, *p* = 0.007). Maternal serum holoTC levels were also positively associated with methylation at these sites (*r* = 0.55 and 0.46, *p* = 0.005 and 0.012). Interestingly, maternal plasma homocysteine levels were positively associated with methylation of CpG1 as well (*r* = 0.94, *p* = 0.001). We also found an inverse association between maternal choline intake and cord blood levels of cortisol, a downstream hormone of CRH (*r* = − 0.36, *P* = 0.021). Maternal holoTC, however, despite the association with higher *CRH* methylation, was not associated with cord cortisol levels (*p* = 0.47). None of the above associations were modified by GDM status.

### Maternal serum folate levels were negatively associated with DNMT expression in the placenta

DNMTs mediate DNA methylation reactions. Previous studies have suggested that methyl nutrients may influence DNMT expression, thereby serving as another mechanism to affect epigenetic modification [27, 30]. We found that maternal serum folate levels were negatively associated with both placental *DNMT1* (*r* = − 0.38, *p* = 0.015) and *DNMT3A* (*r* = − 0.32, *p* = 0.032) expression (Table 5), consistent with the inverse association with *IGF2* DMR0 methylation reported above. Other methyl nutrient intake or blood level was not associated with DNMT expression. GDM status did not modify the above associations.

**Table 5:** Associations between maternal methyl nutrient intake or blood levels and placental *DNMT* expression or cord blood SAM/SAH ratios

	*DNMT1*		*DNMT3A*		Cord SAM/SAH	
	*r*	*p*	R	*p*	*r*	*p*
Intake						
Choline	0.002	0.95	0.074	0.63	0.013	0.91
Betaine	− 0.034	0.88	0.035	0.86	0.13	0.6
Vitamin B_12_	− 0.28	0.055	− 0.17	0.25	− 0.19	0.17
Folate	− 0.07	0.63	− 0.038	0.8	0.087	0.63
Dietary methylation score	− 0.19	0.2	− 0.079	0.55	0.018	0.91
Blood metabolite						
Choline	0.079	0.48	− 0.064	0.78	0.44	0.016
Betaine	0.15	0.31	0.053	0.73	0.089	0.39
Holotranscobalamin	0.02	0.89	0.046	0.78	0.045	0.56
Folate	− 0.38	0.015	− 0.32	0.032	− 0.079	0.58
Homocysteine	0.092	0.53	0.008	0.93	0.14	0.47

*n* = 21 for the GDM group and *n* = 26 for the non-GDM group. Analyzed with generalized linear model adjusted for GDM status, GDM × nutrient intake/blood status interaction, total energy intake, and fetal covariates including sex of neonate, gestational age at birth, and mode of delivery. The correlation coefficient (*r*) was calculated using partial correlation controlling for the variables mentioned above. *DNMT* DNA methyltransferase, *SAM* S-adenosylmethionine, *SAH* S-adenosylhomocysteine

### Maternal plasma choline levels were positively associated with the cord blood SAM/SAH ratio

The SAM/SAH ratio is suggested as a reflection of methylation potential although it should be cautioned that in some tissues this ratio is homeostatic and not responsive to methyl group supplies [24]. We found that maternal plasma choline levels were positively associated with cord blood SAM/SAH (*r* = 0.44, *p* = 0.016) (Table 5). Other methyl nutrient intake/level were not associated with this marker. We were not able to assess placental SAM/SAH ratio due to substantial degradation of SAM in the samples.

## Discussion

In this study, we examined the association of maternal methyl nutrient intake and blood status with placental and fetal epigenetic marks in GDM versus non-GDM pregnancies. We found that maternal methyl nutrient intake and blood level have both positive and negative associations with global and site-specific DNA methylation, which were largely not modified by GDM status. These methylation alterations may have functional consequences such as altered HPA axis activity and fetal growth mediated by the IGF pathway.

Both the placenta and cord blood are fetal derived tissues undergoing substantial developmental changes during pregnancy. Previous studies suggest that they are both susceptible to intrauterine exposures such as maternal nutrition deficiency or excess [34–36] and environmental toxins [37, 38]. In this study, we found that maternal methyl nutrient intakes and blood levels were related to both global and site-specific methylation of multiple CpG loci in the *CRH* promoter and *IGF2* DMR0 of these fetal tissues.

The IGF pathway promotes fetal growth. As an imprinted gene, *IGF2* methylation status is correlated with placental and fetal size [39]. We found that maternal betaine intakes were negatively associated with average methylation of *IGF2* DMR0 in the cord blood. While this inverse relationship between intake of methyl nutrients and methylation may appear contradictory to the model that increasing substrate supply, i.e., methyl groups, would increase DNA methylation, the existing data suggest a more complex relationship between maternal methyl donor supply and offspring tissue methylation.

The inverse relationship between maternal methyl nutrient intake and offspring methylation has also been reported in other studies. For example, in a cohort of 438 pregnant women, folic acid intake before and during pregnancy was associated with lower methylation at the *H19* DMR which regulated *IGF2* expression in the cord blood [40]. Another observational study of 65 maternal-infant pairs also demonstrated that folic acid intake before and during pregnancy was associated with lower *IGF2* methylation in buccal cells of 6-month infants [27]. Our current study suggests that the placenta may serve as a mediator of epigenetic programming. We found that maternal betaine intakes were also inversely related to *IGF2* mRNA expression in the placenta, despite the lack of effect on placental *IGF2* methylation. Placental *IGF2* expression during the fetal period determines placental size and fetal growth [32]. Indeed, we found that maternal betaine intake was inversely correlated with birthweight. As such, our results suggest a potential mechanism that increasing maternal betaine intake may have lowered placental *IGF2* expression, thereby reducing fetal growth as manifested by lower birthweight. This decrease in fetal growth/birth weight may have induced a feedback response in the fetus, lowering fetal cord blood *IGF2* methylation. The Dutch Famine study observed lower *IGF2* methylation in prenatally famine exposed subjects who were at heightened risk of cardiometabolic diseases [41, 42]. Nevertheless, we did not find an association between cord blood IGF2 protein levels and maternal betaine intake in our current study. Therefore, the long-term consequence of lower *IGF2* methylation in infants with higher prenatal betaine intake exposure is not entirely clear.

Serum folate levels were inversely associated with placental *IGF2* methylation, but not *IGF2* gene expression levels in the placenta, underscoring that the association between methyl nutrient status and tissue methylation is indeed more complex than mere substrate provision for methylation. One potential mechanism may be related to the DNMTs, which, as a feedback mechanism, might be inhibited by high folate levels, whereas a folate and methyl deficient diet increased DNMT expression in contrast [43, 44]. Indeed, our study demonstrated that maternal serum folate levels were associated with lower mRNA expression of *DNMT1* and *DNMT3A*, DNA methyltransferases for maintenance and de novo methylation of the genome, respectively. Overall, the higher maternal folate levels in the current study could have inhibited DNMT expression in the placenta, thereby reducing *IGF2* methylation.

Methyl nutrient intakes and blood levels were also associated with epigenetic modifications of the HPA axis, which mediates stress response [45]. In the HPA axis, CRH is released by the hypothalamus, which triggers the release of adrenocorticotropic hormone (ACTH) that further promotes the release of cortisol. Chronic elevation of cortisol is associated with increased risks of cardiometabolic diseases and mental and immune issues in the long term [45]. We previously reported the levels of cord blood cortisol in this cohort [31] and this time we found that maternal choline intake was associated with higher cord blood *CRH* methylation and lower cortisol levels in the cord blood. This seems to be consistent with the mechanism that a higher maternal choline intake increases methyl group supply for cord *CRH* methylation, thereby dampening the expression and activity of the HPA axis in the fetus. These results corroborated a prior RCT where a higher maternal choline intake in the third trimester of pregnancy increased *CRH* methylation and decreased its expression in the placenta [30]. Since placental *CRH* is able to enter cord blood, the less CRH transport from the placenta to the cord blood resulted in lower cortisol levels in the cord blood in the RCT [30]. Overall, these results highlight the importance of maternal choline intake for HPA axis epigenetic regulation of children.

In this study, methyl nutrient levels in maternal blood were also related to global placental DNA methylation. Specifically, both holoTC and homocysteine levels were inversely related to placental DNA methylation. Homocysteine is formed after methylation reactions and a high homocysteine level in the blood may indicate methyl nutrient deficiency. Its association with placental DNA hypomethylation was also reported in previous human studies [46, 47]. It is surprising that high holoTC, which indicates functional vitamin B_12_ levels, was also negatively correlated with placental DNA methylation.

Half of the participants in this study have GDM. GDM has been demonstrated to alter both placental and cord blood epigenome, as well as methyl nutrient status in prior studies [48–50]. In this study, we found that maternal plasma choline levels were higher in GDM versus non-GDM women. The increase in choline levels in case of cardiometabolic stress was seen in previous studies [51], supporting that blood choline may be an indicator of metabolic stress. We also found that the diet of participants with GDM was more concentrated with choline, although total choline intake did not differ due to lower caloric consumption of women with GDM. It is not clear whether the choice of a choline-rich diet was a result of how women with GDM modified their diets because of the disease, or a reflection of dietary habit before GDM development, since dietary recalls were collected after GDM diagnosis. Total folate intake was lower in the GDM versus control group, yet such a difference was eliminated after accounting for total energy intake, possibly reflecting the lower folate fortified grain intake in women with GDM as a strategy to control their blood glucose. *CRH* methylation in the cord blood was also higher in GDM versus non-GDM, suggesting its susceptibility to modifications by cardiometabolic stressors, like GDM in this case. Overall, contrary to what we hypothesized the association between maternal methyl nutrient intake or status and placental and cord DNA methylation were not modified by GDM status, except that homocysteine levels were positively associated with cord blood global DNA methylation in non-GDM but not GDM pregnancies.

One limitation of this study is the lack of nutrition supplement data. Therefore, total intake of methyl nutrients from both diet and supplement cannot be calculated. Nevertheless, since previous studies that examined the supplement of methyl nutrients reported varied associations with epigenetic marks in infants and children [27–30, 52, 53], this study provides novel insights regarding the interaction between maternal methyl nutrient supply and GDM status in modifying the fetal epigenome in humans. It should be noted that most maternal methyl nutrients were not related to fetal global DNA methylation. The associations between maternal methyl nutrients and fetal *IGF2* and *CRH* methylation were also not universal. These non-significant findings could be partly attributed to the relatively small sample size that limits the statistical power to detect subtle differences in methylation levels. In addition, DNA methylation is influenced by various factors besides maternal methyl nutrient supply, such as other intrauterine exposures like environmental toxins and medication use, genetic variations, and even seasonality, as well as their interactions that provide further complexity in shaping the fetal epigenome [2]. Moreover, developmental stages of the fetus and timing of sample collection may also lead to variability in DNA methylation patterns and differential susceptibility to intrauterine exposures [24]. As such, future studies with a larger sample size, incorporating other intrauterine factors, and including dynamic screening of the DNA methylation landscape across developmental stages will provide more comprehensive understanding of the associations among maternal methyl nutrient intake, GDM, and fetal DNA methylation and birth outcomes.

## Conclusions

This study demonstrates that maternal betaine intake during pregnancy was inversely associated with *IGF2* DMR0 methylation, while maternal choline intake was positively associated with *CRH* promoter methylation in the cord blood. These associations may have implications in the lower birth weight and stress hormone cortisol levels observed in the infants with higher maternal intakes of these nutrients, respectively. GDM status may affect the levels of methyl nutrients (e.g., choline) and DNA methylation (e.g., *CRH*), but did not modify the association between maternal methyl nutrients and fetal epigenetic marks.

## Methods

### Study population and study procedures

Pregnant women (*n* = 76) with or without GDM who attended the prenatal clinic at the State University of New York (SUNY) Downstate Health Sciences University in Brooklyn, NY, USA, were recruited to participate in the study from September 2016 to June 2019. Detailed methodology has been published previously [31]. Inclusion criteria included English-speaking, over 21 years of age, gestational age between 25 and 33 weeks, and singleton pregnancy. Exclusion criteria included pre-pregnancy diabetes, cardiovascular conditions including hypercholesterolemia and hypertension that require medical treatment, kidney disease, and liver disease prior to pregnancy. At enrollment, each participant completed a baseline questionnaire which included demographic and medical information. Three 24-h dietary recalls, two on weekdays and one on a weekend day, were obtained from each participant by a trained research assistant via phone following the baseline visit [54, 55]. Dietary data was analyzed with the Nutrition Data System for Research (NDSR) software to analyze nutrient intake. Average daily intakes were calculated as the average daily consumption of nutrients over the 3 days of dietary recalls. Overall diet quality was assessed using the Healthy Eating Index (HEI) 2015 [56–58]. We excluded dietary recalls with extremely low (< 500 kcal/day) or high (> 5000 kcal/day) total energy intake in data analysis. We also derived a DMS that reflected each participant’s overall methyl nutrient consumption. Specifically, we divided the dietary intake of betaine, choline, folate, and vitamin B_12_ into quartiles, assigning scores 1–4 from the lowest to the highest quartile of intake. The composite DMS is the sum of quartile ranking scores of all four nutrients [59, 60].

The participants were instructed to fast overnight for at least 8 h at enrollment (week 25–33 of gestation), then 10 mL of blood was drawn to retrieve plasma. Immediately after delivery, cord venous blood was collected to retrieve plasma. Full thickness placenta biopsies (0.5 × 0.5 cm) were retrieved at the center of four virtually divided quadrants of the placental disk and pooled for analysis [30]. These pooled placental samples were homogenized in liquid nitrogen. All samples were stored at − 80 °C until analysis. Obstetric and birth outcome information including neonate sex, mode of delivery, and birth weight was collected through medical chart review.

The study protocol was reviewed and approved by both the City University of New York (CUNY) Institutional Review Board (IRB) (approval ID: 2016-0331) and the SUNY Downstate Health Sciences University IRB (approval ID: 816,786). Written informed consent was obtained from each participant before participation in the study.

### Analytical measurements

Choline metabolites were measured by liquid chromatography mass spectrometry liquid chromatography-mass spectrometry (LC–MS)/MS using published methods [61]. Maternal serum folate was measured with the ALPCO folic acid vitamin B9 microbiological test kit (ALPCO, Salem, NH, USA). Maternal plasma homocysteine was measured by the homocysteine ELISA kit (Cell Biolabs, Inc. San Diego, CA, USA). Maternal serum transcobalamin levels were measured with the human holoTC ELISA kit (MyBioSource, Inc., San Diego, CA, USA). Cord blood plasma *S*-adenosylmethionine (SAM) and *S*-adenosylhomocysteine (SAH) levels were measured with the SAM and SAH ELISA Combo kit (Cell BIolabs, Inc) and the SAM/SAH ratio was derived from the ratio of the two metabolites’ concentrations. Cord plasma IGF2 levels were also measured with ELISA (ALPCO) following manufacturer’s instructions.

DNA was extracted from the placenta and cord blood samples using the GeneJET Genomic DNA Purification Kit (Thermo Fisher, Suwanee, GA, USA) following manufacturer’s instructions. Global methylation was assessed after nuclease P1, alkaline phosphatase, and phosphodiesterase (Sigma-Aldrich, St. Louis, MO) trienzyme treatment of the DNA samples, followed with measurements using the DNA methylation ELISA kit (Cayman Chemical, Ann Arbor, Michigan, USA) following manufacturer’s instructions. Site-specific methylation of *CRH* and *IGF2* was assessed using the bisulfite method [30]. Briefly, DNA was bisulfite treated using the EZ-DNA methylation kit (Zymo, Irvine, CA, USA). Thereafter, PCR was conducted on sequences located in the *CRH* promoter (chr8:67,090,692–67,091,132) and the *IGF2* DMR (chr11:2,169,459–2,169,796) using a published protocol and primers (30). Previous literature has demonstrated that hypermethylation of the *CRH* promoter can lead to lower expression [30]. The *IGF2* DMR is homologous to the mouse *Igf2* DMR0 that locates upstream of the promoter 3 and is maternally methylated. Its loss of methylation is associated with colorectal cancers and Wilm’s tumors [33]. Sequence amplified and CpG loci included in the region were also previously published [30]. Targeted DNA methylation analysis was carried out at the Epigenetic core facility of City University of New York (CUNY) Advanced Science Research Center (ASRC) using the Epityper MassArray instrument [62]. Only CpG sites with over 5% methylation and less than 25% missing data were included in the final analysis. Real-time quantitative PCR was conducted on the placental tissue using a published SYBR green based method [30]. RNA transcripts assessed include DNA methyltransferase 1 (*DNMT1*) and *DNMT3A* using published primers [30].

### Statistical analyses

To compare methyl nutrient intake, blood status and DNA methylation between GDM and non-GDM, an ANCOVA model was used with GDM status as a fixed factor (Model 1). To account for potential demographic and obstetric confounders (Model 2), maternal age, parity, race/ethnicity, and maternal education levels were entered as covariates as previous studies suggest that maternal demographic characteristics could lead to differences in the epigenome [63, 64]. In the full model (Model 3), pre-pregnancy BMI (which influences energy requirement) and total energy intake were entered as covariates for dietary-intake-related analyses in addition to covariates in model 2. Model 3 represents the nutrient density in the diet. For cord blood and placenta epigenetic biomarkers, additional covariates adjusted for in model 2 and 3 included neonate sex and gestational age at birth since these characteristics are related to differential fetal growth and IGF pathway methylation [65, 66], as well as mode of delivery since it may affect stress response of infants [67].

To assess the association among maternal methyl nutrient intake/blood level and maternal/cord epigenetic biomarkers, the generalized linear model was used. The nutrient intake/blood level was entered as the independent variable, and the epigenetic marker as the dependent variable, adjusted for GDM status, GDM × nutrient intake/blood status interaction, total energy intake, and fetal covariates including sex of neonate, gestational age at birth, and mode of delivery. The correlation coefficient (*r*) was calculated using partial correlation controlling for the variables mentioned above. If there was a trend of interaction between GDM status and nutrient intake/blood status in their correlation with an epigenetic outcome (*p* < 0.1), we conducted a secondary regression analysis stratifying the data by GDM status. For average methylation of *CRH* and *IGF2*, a generalized linear mixed model was used with methylation of CpGs as the independent variable, and the dependent variables noted above plus the individual CpG unit as fixed factors and participant ID as a random factor. Data that were not normally distributed (i.e., SAM/SAH ratios) were log-transformed before analyses. Data were presented as mean ± standard deviation (SD). A *p* value of < 0.05 was considered significant. Data were analyzed with SPSS software (version 24, IBM Inc., Armonk, NY, USA).

### Supplementary Information

**Additional file 1**. **Table S1**: Baseline and birth outcome characteristics of participants

**Additional file 2**. **Table S2**: Associations between maternal methyl nutrient intake or blood levels and IGF2 DMR0 CpG sites DNA methylation in the placenta and cord blood

**Additional file 3**. **Table S3**: Associations between maternal methyl nutrient intake or blood levels and CRH promoter CpG sites DNA methylation in the placenta and cord blood

## Data Availability

The datasets supporting the conclusions of this article are available upon request.

## References

[1] Allis CD, Jenuwein T (2016). The molecular hallmarks of epigenetic control. Nat Rev Genet.

[2] James P, Sajjadi S, Tomar AS, Saffari A, Fall CHD, Prentice AM (2018). Candidate genes linking maternal nutrient exposure to offspring health via DNA methylation: a review of existing evidence in humans with specific focus on one-carbon metabolism. Int J Epidemiol.

[3] Chen T, Liu HX, Yan HY, Wu DM, Ping J (2016). Developmental origins of inflammatory and immune diseases. Mol Hum Reprod.

[4] Chu SY, Callaghan WM, Kim SY, Schmid CH, Lau J, England LJ (2007). Maternal obesity and risk of gestational diabetes mellitus. Diabetes Care.

[5] Atègbo JM, Grissa O, Yessoufou A, Hichami A, Dramane KL, Moutairou K (2006). Modulation of adipokines and cytokines in gestational diabetes and macrosomia. J Clin Endocrinol Metab.

[6] Franks PW, Looker HC, Kobes S, Touger L, Tataranni PA, Hanson RL (2006). Gestational glucose tolerance and risk of type 2 diabetes in young Pima Indian offspring. Diabetes.

[7] Page KA, Romero A, Buchanan TA, Xiang AH (2014). Gestational diabetes mellitus, maternal obesity, and adiposity in offspring. J Pediatr.

[8] Kelstrup L, Hjort L, Houshmand-Oeregaard A, Clausen TD, Hansen NS, Broholm C (2016). Gene expression and DNA methylation of PPARGC1A in muscle and adipose tissue from adult offspring of women with diabetes in pregnancy. Diabetes.

[9] Hansen NS, Strasko KS, Hjort L, Kelstrup L, Houshmand-Øregaard A, Schrölkamp M (2017). Fetal hyperglycemia changes human preadipocyte function in adult life. J Clin Endocrinol Metab.

[10] Houshmand-Oeregaard A, Hansen NS, Hjort L, Kelstrup L, Broholm C, Mathiesen ER (2017). Differential adipokine DNA methylation and gene expression in subcutaneous adipose tissue from adult offspring of women with diabetes in pregnancy. Clin Epigenet.

[11] Allard C, Desgagné V, Patenaude J, Lacroix M, Guillemette L, Battista MC (2015). Mendelian randomization supports causality between maternal hyperglycemia and epigenetic regulation of leptin gene in newborns. Epigenetics.

[12] Su R, Wang C, Feng H, Lin L, Liu X, Wei Y (2016). Alteration in expression and methylation of IGF2/H19 in placenta and umbilical cord blood are associated with macrosomia exposed to intrauterine hyperglycemia. PLoS One.

[13] Zhang Q, Su R, Qin S, Wei Y (2022). High glucose increases IGF-2/H19 expression by changing DNA methylation in HTR8/SVneo trophoblast cells. Placenta.

[14] Tien Nguyen S, Bui Minh T, Trung Dinh H, Dinh Le T, Phi Thi Nguyen N, Tran TTH (2023). Relationship between maternal serum cortisol and maternal insulin resistance and fetal ultrasound characteristics in gestational diabetes mellitus. Diabetes Metab Syndr Obes.

[15] Fan F, Zou Y, Zhang Y, Ma X, Zhang J, Liu C (2018). The relationship between maternal anxiety and cortisol during pregnancy and birth weight of chinese neonates. BMC Pregnancy Childbirth.

[16] Sheng JA, Bales NJ, Myers SA, Bautista AI, Roueinfar M, Hale TM (2020). The hypothalamic-pituitary-adrenal axis: development, programming actions of hormones, and maternal-fetal interactions. Front Behav Neurosci.

[17] O'Donnell K, O'Connor TG, Glover V (2009). Prenatal stress and neurodevelopment of the child: focus on the HPA axis and role of the placenta. Dev Neurosci.

[18] Xiong F, Zhang L (2013). Role of the hypothalamic–pituitary–adrenal axis in developmental programming of health and disease. Front Neuroendocrinol.

[19] Poston L, Bell R, Croker H, Flynn AC, Godfrey KM, Goff L (2015). Effect of a behavioural intervention in obese pregnant women (the UPBEAT study): a multicentre, randomised controlled trial. Lancet Diabetes Endocrinol.

[20] Sagedal LR, Øverby NC, Bere E, Torstveit MK, Lohne-Seiler H, Småstuen M (2017). Lifestyle intervention to limit gestational weight gain: the Norwegian Fit for Delivery randomised controlled trial. BJOG.

[21] Han S, Crowther CA, Middleton P, Heatley E (2013). Different types of dietary advice for women with gestational diabetes mellitus. Cochrane Database Syst Rev.

[22] Bain E, Crane M, Tieu J, Han S, Crowther CA, Middleton P (2015). Diet and exercise interventions for preventing gestational diabetes mellitus. Cochrane Database Syst Rev.

[23] Catalano PM, Shankar K (2017). Obesity and pregnancy: mechanisms of short term and long term adverse consequences for mother and child. BMJ.

[24] Korsmo HW, Jiang X (2021). One carbon metabolism and early development: a diet-dependent destiny. Trends Endocrinol Metab.

[25] Waterland RA, Travisano M, Tahiliani KG, Rached MT, Mirza S (2008). Methyl donor supplementation prevents transgenerational amplification of obesity. Int J Obes (Lond).

[26] Waterland RA, Lin JR, Smith CA, Jirtle RL (2006). Post-weaning diet affects genomic imprinting at the insulin-like growth factor 2 (Igf2) locus. Hum Mol Genet.

[27] Pauwels S, Ghosh M, Duca RC, Bekaert B, Freson K, Huybrechts I (2017). Maternal intake of methyl-group donors affects DNA methylation of metabolic genes in infants. Clin Epigenet.

[28] Steegers-Theunissen RP, Obermann-Borst SA, Kremer D, Lindemans J, Siebel C, Steegers EA (2009). Periconceptional maternal folic acid use of 400 microg per day is related to increased methylation of the IGF2 gene in the very young child. PLoS One.

[29] Haggarty P, Hoad G, Campbell DM, Horgan GW, Piyathilake C, McNeill G (2013). Folate in pregnancy and imprinted gene and repeat element methylation in the offspring. Am J Clin Nutr.

[30] Jiang X, Yan J, West AA, Perry CA, Malysheva OV, Devapatla S (2012). Maternal choline intake alters the epigenetic state of fetal cortisol-regulating genes in humans. FASEB J.

[31] Jack-Roberts C, Maples P, Kalkan B, Edwards K, Gilboa E, Djuraev I (2020). Gestational diabetes status and dietary intake modify maternal and cord blood allostatic load markers. BMJ Open Diabetes Res Care.

[32] Constância M, Hemberger M, Hughes J, Dean W, Ferguson-Smith A, Fundele R (2002). Placental-specific IGF-II is a major modulator of placental and fetal growth. Nature.

[33] Murrell A, Heeson S, Cooper WN, Douglas E, Apostolidou S, Moore GE (2004). An association between variants in the IGF2 gene and Beckwith–Wiedemann syndrome: interaction between genotype and epigenotype. Hum Mol Genet.

[34] Belkacemi L, Nelson DM, Desai M, Ross MG (2010). Maternal undernutrition influences placental-fetal development. Biol Reprod.

[35] Shen WB, Ni J, Yao R, Goetzinger KR, Harman C, Reece EA (2022). Maternal obesity increases DNA methylation and decreases RNA methylation in the human placenta. Reprod Toxicol.

[36] Martin CL, Jima D, Sharp GC, McCullough LE, Park SS, Gowdy KM (2019). Maternal pre-pregnancy obesity, offspring cord blood DNA methylation, and offspring cardiometabolic health in early childhood: an epigenome-wide association study. Epigenetics.

[37] Nakamura A, François O, Lepeule J (2021). Epigenetic alterations of maternal tobacco smoking during pregnancy: a narrative review. Int J Environ Res Public Health.

[38] Ouidir M, Mendola P, Buck Louis GM, Kannan K, Zhang C, Tekola-Ayele F (2020). Concentrations of persistent organic pollutants in maternal plasma and epigenome-wide placental DNA methylation. Clin Epigenet.

[39] St-Pierre J, Hivert MF, Perron P, Poirier P, Guay SP, Brisson D (2012). IGF2 DNA methylation is a modulator of newborn’s fetal growth and development. Epigenetics.

[40] Hoyo C, Murtha AP, Schildkraut JM, Jirtle RL, Demark-Wahnefried W, Forman MR (2011). Methylation variation at IGF2 differentially methylated regions and maternal folic acid use before and during pregnancy. Epigenetics.

[41] Tobi EW, Lumey LH, Talens RP, Kremer D, Putter H, Stein AD (2009). DNA methylation differences after exposure to prenatal famine are common and timing- and sex-specific. Hum Mol Genet.

[42] Tobi EW, Goeman JJ, Monajemi R, Gu H, Putter H, Zhang Y (2014). DNA methylation signatures link prenatal famine exposure to growth and metabolism. Nat Commun.

[43] Ghoshal K, Li X, Datta J, Bai S, Pogribny I, Pogribny M (2006). A folate- and methyl-deficient diet alters the expression of DNA methyltransferases and methyl CpG binding proteins involved in epigenetic gene silencing in livers of F344 rats. J Nutr.

[44] Mahajan A, Sapehia D, Thakur S, Mohanraj PS, Bagga R, Kaur J (2019). Effect of imbalance in folate and vitamin B12 in maternal/parental diet on global methylation and regulatory miRNAs. Sci Rep.

[45] Smith SM, Vale WW (2006). The role of the hypothalamic–pituitary–adrenal axis in neuroendocrine responses to stress. Dialogues Clin Neurosci.

[46] Park BH, Kim YJ, Park JS, Lee HY, Ha EH, Min JW (2005). Folate and homocysteine levels during pregnancy affect DNA methylation in human placenta. J Prev Med Public Health.

[47] Kim JM, Hong K, Lee JH, Lee S, Chang N (2009). Effect of folate deficiency on placental DNA methylation in hyperhomocysteinemic rats. J Nutr Biochem.

[48] Houde AA, St-Pierre J, Hivert MF, Baillargeon JP, Perron P, Gaudet D (2014). Placental lipoprotein lipase DNA methylation levels are associated with gestational diabetes mellitus and maternal and cord blood lipid profiles. J Dev Orig Health Dis.

[49] Nomura Y, Lambertini L, Rialdi A, Lee M, Mystal EY, Grabie M (2014). Global methylation in the placenta and umbilical cord blood from pregnancies with maternal gestational diabetes, preeclampsia, and obesity. Reprod Sci.

[50] Finer S, Mathews C, Lowe R, Smart M, Hillman S, Foo L (2015). Maternal gestational diabetes is associated with genome-wide DNA methylation variation in placenta and cord blood of exposed offspring. Hum Mol Genet.

[51] Wang Z, Klipfell E, Bennett BJ, Koeth R, Levison BS, Dugar B (2011). Gut flora metabolism of phosphatidylcholine promotes cardiovascular disease. Nature.

[52] Caffrey A, Irwin RE, McNulty H, Strain JJ, Lees-Murdock DJ, McNulty BA (2018). Gene-specific DNA methylation in newborns in response to folic acid supplementation during the second and third trimesters of pregnancy: epigenetic analysis from a randomized controlled trial. Am J Clin Nutr.

[53] Pauwels S, Ghosh M, Duca RC, Bekaert B, Freson K, Huybrechts I (2017). Dietary and supplemental maternal methyl-group donor intake and cord blood DNA methylation. Epigenetics.

[54] Harnack L, Stevens M, Van Heel N, Schakel S, Dwyer JT, Himes J (2008). A computer-based approach for assessing dietary supplement use in conjunction with dietary recalls. J Food Compost Anal.

[55] Arab L, Tseng CH, Ang A, Jardack P (2011). Validity of a multipass, web-based, 24-h self-administered recall for assessment of total energy intake in blacks and whites. Am J Epidemiol.

[56] Miller PE, Mitchell DC, Harala PL, Pettit JM, Smiciklas-Wright H, Hartman TJ (2011). Development and evaluation of a method for calculating the healthy eating index-2005 using the nutrition data system for research. Public Health Nutr.

[57] Guenther PM, Casavale KO, Reedy J, Kirkpatrick SI, Hiza HA, Kuczynski KJ (2013). Update of the healthy eating index: HEI-2010. J Acad Nutr Diet.

[58] Panizza CE, Shvetsov YB, Harmon BE, Wilkens LR, Le Marchand L, Haiman C (2018). Testing the predictive validity of the healthy eating index-2015 in the multiethnic cohort: is the score associated with a reduced risk of all-cause and cause-specific mortality?. Nutrients.

[59] Poursalehi D, Lotfi K, Mirzaei S, Asadi A, Akhlaghi M, Saneei P (2022). Association between methyl donor nutrients and metabolic health status in overweight and obese adolescents. Sci Rep.

[60] Marley AR, Fan H, Hoyt ML, Anderson KE, Zhang J (2018). Intake of methyl-related nutrients and risk of pancreatic cancer in a population-based case-control study in Minnesota. Eur J Clin Nutr.

[61] Yan J, Jiang X, West AA, Perry CA, Malysheva OV, Devapatla S (2012). Maternal choline intake modulates maternal and fetal biomarkers of choline metabolism in humans. Am J Clin Nutr.

[62] Korsmo HW, Dave B, Trasino S, Saxena A, Liu J, Caviglia JM (2022). Maternal choline supplementation and high-fat feeding interact to influence DNA methylation in offspring in a time-specific manner. Front Nutr.

[63] Leimert K, Olson D (2020). Racial disparities in pregnancy outcomes: genetics, epigenetics, and allostatic load. Curr Opin Physio.

[64] Markunas CA, Wilcox AJ, Xu Z, Joubert BR, Harlid S, Panduri V (2016). Maternal age at delivery is associated with an epigenetic signature in both newborns and adults. PLoS One.

[65] Bozack AK, Colicino E, Just AC, Wright RO, Baccarelli AA, Wright RJ (2022). Associations between infant sex and DNA methylation across umbilical cord blood, artery, and placenta samples. Epigenetics.

[66] Zhang S, Zhai G, Wang J, Shi W, Zhang R, Chen C (2015). IGF-II expression and methylation in small for gestational age infants. J Pediatr Endocrinol Metab.

[67] Martinez LD, Glynn LM, Sandman CA, Wing DA, Davis EP (2020). Cesarean delivery and infant cortisol regulation. Psychoneuroendocrinology.

