# Supplementary material for: Associations between nutrients in one-carbon metabolism and fetal DNA methylation in pregnancies with or without gestational diabetes mellitus
Source: Clin Epigenetics. 2023 Aug 26;15:137. doi: 10.1186/s13148-023-01554-1 (PMC10464204; doi:10.1186/s13148-023-01554-1)
Supplement: Supplementary file 1 — Additional file 1. Table S1: Baseline and birth outcome characteristics of participants [file 13148_2023_1554_MOESM1_ESM.docx]

Table S1. Baseline and birth outcome characteristics of participants^1^

|  | **GDM (n = 40)** | **Control (n = 36)** | ***p*** |
| --- | --- | --- | --- |
| Maternal age (year) | 32.6 ± 5.2 | 32.5 ± 5.6 | 0.92 |
| Parity |  |  | 0.98 |
| Nulliparous | 9 (23%) | 8 (22%) |  |
| Multiparous | 31 (77%) | 28 (78%) |  |
| Race/ethnicity |  |  | 0.78 |
| Non-Hispanic white | 0 (0%) | 1 (2.7%) |  |
| Non-Hispanic black | 33 (82.5%) | 32 (89.2%) |  |
| Hispanic white | 4 (10%) | 1 (2.7%) |  |
| Asian | 2 (5%) | 0 (0%) |  |
| Other/mixed race | 1 (2.5%) | 2 (5.4%) |  |
| Education level |  |  | 0.33 |
| ≤ High school | 29 (73%) | 23 (64%) |  |
| ≥ Some college | 11 (27%) | 13 (36%) |  |
| Pre-pregnancy BMI (kg/m^2^) | 33.8 ± 7.2 | 28.3 ± 7.5 | < 0.01 |
| Gestational weight gain (kg) | 11.6 ± 11.0 | 11.1 ± 8.0 | 0.83 |
| Gestational age (week) | 37.3 ± 2.0 | 38.9 ± 1.5 | < 0.01 |
| Cesarean section (n,%) | 24 (60%) | 17 (47%) | 0.46 |
| Pre-term delivery (n,%) | 11 (27.5%) | 2 (5.6%) | < 0.01 |
| Sex of neonate (male) (n, %) | 16 (40%) | 16 (44%) | 0.69 |
| Birth weight (g) | 3339 ± 756 | 3097 ± 468 | 0.12 |

^1^ Analyzed with student’s t test for continuous variables and χ-square test or Fisher’s exact test (for the race/ethnicity variable) for categorical variables. Values are mean ± SD.
