# Supplementary material for: Associations between nutrients in one-carbon metabolism and fetal DNA methylation in pregnancies with or without gestational diabetes mellitus
Source: Clin Epigenetics. 2023 Aug 26;15:137. doi: 10.1186/s13148-023-01554-1 (PMC10464204; doi:10.1186/s13148-023-01554-1)
Supplement: Supplementary file 3 — Additional file 3. Table S3: Associations between maternal methyl nutrient intake or blood levels and CRH promoter CpG sites DNA methylation in the placenta and cord blood [file 13148_2023_1554_MOESM3_ESM.docx]

Table S3. Associations between maternal methyl nutrient intake or blood levels and *CRH* promoter CpG sites DNA methylation in the placenta and cord blood

|  | CpG1 | CpG2 | CpG4 | CpG8 | CpG9 | CpG10 |
| --- | --- | --- | --- | --- | --- | --- |
|  | r (p) | r (p) | r (p) | r (p) | r (p) | r (p) |
| *Placenta* |  |  |  |  |  |  |
| Choline intake | -0.28 (0.11) | -0.13 (0.46) | 0.046 (0.83) | 0.12 (0.46) | 0.13 (0.29) | -0.016 (0.95) |
| Betaine intake | 0.096 (0.44) | -0.12 (0.51) | 0.057 (0.83) | 0.34 (0.043) | -0.08 (0.66) | -0.37 (0.025) |
| Vitamin B_12_ intake | -0.049 (0.70) | -0.069 (0.69) | -0.004 (0.97) | 0.36 (0.014) | 0.16 (0.25) | -0.23 (0.14) |
| Folate intake | 0.056 (0.71) | -0.006 (0.97) | 0.12 (0.48) | 0.24 (0.13) | 0.24 (0.12) | -0.20 (0.21) |
| Dietary methylation score | -0.072 (0.68) | -0.14 (0.42) | 0.21 (0.23) | 0.34 (0.026) | 0.10 (0.43) | -0.29 (0.059) |
| Maternal plasma choline | 0.10 (0.59) | -0.087 (0.47) | -0.092 (0.55) | 0.043 (0.69) | 0.095 (0.53) | 0.22 (0.20) |
| Maternal plasma betaine | 0.28 (0.072) | -0.075 (0.67) | 0.19 (0.28) | 0.38 (0.009) | 0.19 (0.21) | 0.056 (0.70) |
| Maternal serum holotranscobalamin | -0.21 (0.21) | -0.38 (0.011) | -0.18 (0.30) | 0.081 (0.59) | 0.17 (0.27) | 0.11 (0.49) |
| Maternal serum folate | -0.025 (0.94) | -0.33 (0.033) | -0.11 (0.56) | 0.043 (0.89) | 0.054 (0.68) | -0.24 (0.18) |
| Maternal plasma homocysteine | -0.19 (0.30) | -0.30 (0.079) | -0.37 (0.062) | 0.034 (0.75) | 0.091 (0.47) | 0.16 (0.36) |
| *Cord* |  |  |  |  |  |  |
| Choline intake | 0.55 (0.008) | 0.46 (0.007) |  | -0.029 (0.89) | 0.34 (0.068) |  |
| Betaine intake | -0.47 (0.09) | -0.12 (0.54) |  | 0.16 (0.46) | -0.065 (0.75) |  |
| Vitamin B_12_ intake | 0.40 (0.24) | 0.025 (0.94) |  | 0.061 (0.72) | -0.1 (0.59) |  |
| Folate intake | 0.32 (0.22) | 0.075 (0.71) |  | 0.17 (0.38) | 0.025 (0.89) |  |
| Dietary methylation score | 0.49 (0.058) | 0.071 (0.64) |  | 0.076 (0.69) | 0.059 (0.79) |  |
| Maternal plasma choline | 0.16 (0.89) | 0.10 (0.45) |  | -0.36 (0.057) | 0.23 (0.26) |  |
| Maternal plasma betaine | 0.067 (0.97) | 0.14 (0.47) |  | -0.038 (0.85) | 0.17 (0.36) |  |
| Maternal serum holotranscobalamin | 0.55 (0.005) | 0.46 (0.012) |  | 0.27 (0.16) | -0.11 (0.53) |  |
| Maternal serum folate | 0.085 (0.73) | 0.15 (0.39) |  | 0.005 (0.91) | -0.002 (0.98) |  |
| Maternal plasma homocysteine | 0.94 (0.001) | 0.23 (0.10) |  | -0.16 (0.40) | 0.12 (0.43) |  |

n = 21 for the GDM group and n = 26 for the non-GDM group. Analyzed with generalized linear model adjusted for GDM status, GDM × nutrient intake/blood status interaction, total energy intake, and fetal covariates including sex of neonate, gestational age at birth, and mode of delivery. The correlation coefficient (r) was calculated using partial correlation controlling for the variables mentioned above. CRH: corticotropin-releasing hormone
